# Exploring the ambiguity in the anatomical terminology among Dental professionals

**DOI:** 10.1186/s12909-024-05878-1

**Published:** 2024-08-22

**Authors:** Kanet Chotvorrarak, Tawepong Arayapisit, Lalida Matthayomnan, Panupong Thampibul, Piyada Gaewkhiew

**Affiliations:** 1 Department of Operative Dentistry and Endodontics, Faculty of Dentistry, Mahidol University, Thailand, 10400 Thailand; 2 Department of Anatomy, Faculty of Dentistry, Mahidol University, Thailand, 10400 Thailand; 3 Dr Sea Dental Clinic, Rayong, Thailand; 4 Panomsarakarm Hospital, Chachoengsao, Thailand; 5 Department of Community Dentistry, Faculty of Dentistry, Mahidol University, Thailand, 10400 Thailand

**Keywords:** Anatomy, Dental students, Dental education, Medical education

## Abstract

**Background:**

Anatomical terms in medical literature have been used with varying meanings, leading to confusion in clinical practice. This study aims to investigate the ambiguity of anatomical terms in clinical dentistry.

**Methods:**

Dentists who have undergone specialised training with at least one year of clinical experience were recruited to participate in the study. They were requested to localize specific terms on a skull and provide explanations based on their experience or opinion. All data were recorded, and then descriptive statistics were used for analysis.

**Results:**

Seventy-eight participating dentists gave their consent and were eligible to study. For each anatomical term presented to dentists at least two meanings were provided, with some terms having up to eight interpretations. While most meanings were consistent with medical or dental literature, some responses revealed new interpretations not documented in textbooks.

**Conclusions:**

Dentists expressed anatomical terms with diverse meanings, possibly influenced by their various subspecialties. It is crucial to acknowledge this variability to prevent confusion. Emphasizing the consistent use of anatomical terms among dental professionals in the future is essential.

**Supplementary Information:**

The online version contains supplementary material available at 10.1186/s12909-024-05878-1.

## Background

Anatomical terminology constitutes a specialized collection of terms employed to delineate the intricate structures within the human body, forming the foundational basis for communication across various healthcare disciplines, including dentistry. The vast majority of these terms are specific and discernible exclusively to professionals, making them uncommon in daily communication [1]. The history of anatomical terms can be traced back over 2500 years. From ancient Greece to the present day, these terms have developed in various regions over time, driven by evolving usage needs. This diversification has led to an increase in the quantity of terms and a greater degree of complexity. Systematic efforts have been made to organize these terms, resulting in the development of the International Anatomical Terminology. Its first edition was recognized as Nomina Anatomica. Subsequent revisions and refinements have led to the current standard lexicon known as Terminologia Anatomica (TA) [2].

Previous studies on discrepancies in anatomical terminology [3–6] have shown that many anatomical terms also have synonyms. For example, the term ‘pineal gland’ has 16 synonyms, including pineal body, epiphysis, and parietal eye [1, 3]. Therefore, this might intensify the academic burden on students, compelling them to commit a greater extent of these terms to memory [3, 4].

Consequently, each newly emerging field endeavoured to formulate novel anatomical terminology tailored to its specific communication needs [5]. New sets of terminology have emerged. Some of these terms were not found in TA, such as the ‘retromolar triangle’ whereas the other terms were synonyms of TA terms, or even terms identical to TA but with new meanings. For instance, the term ‘internal oblique line of mandible’ is synonymous with the TA term ‘mylohyoid line,’ which refers to the attachment of the mylohyoid muscle [6]. Additionally, as a non-TA term, it denotes a bony ridge on the inner aspect of the mandibular ramus, extending from the coronoid process to the last molar, serving as the insertion point for the deep tendon of the temporalis muscle [7, 8].

Initially, anatomical terms were systematically compiled to facilitate communication within the healthcare community. However, with the creation of these terms, encompassing not only existing TA terms but also new terminologies, synonyms, and homonyms, there has been a notable increase in the overall volume of terms [9, 10] heightening the risk of confusion in both communication and interpretation [4, 11]. Undoubtedly, it imposes a substantial burden on learners in the field.

Dentistry, similar to medical professions, constitutes a complex and ever-evolving domain requiring a profound understanding of human anatomy. Dental professionals rely extensively on anatomical terminology to communicate effectively, diagnose oral health conditions, and administer appropriate treatments. However, not only anatomical terms but also specific dental jargon, such as “centric occlusion,” lacks consensus among academics. This lack of agreement poses challenges for dental students in comprehending and applying inconsistent terminologies found across various learning resources, ultimately leading to confusion in knowledge acquisition [12]. Furthermore, inconsistent terminology might complicate literature reviews and scientific discussions, potentially impeding advancements in dental research.

Nevertheless, this matter has solely been addressed within academic literature and has not been thoroughly investigated concerning the confusion related to anatomical terms in the clinical setting [13]. Therefore, this study aimed to explore and gather evidence to demonstrate the ambiguity surrounding the utilization of anatomical terms among dental practitioners, thereby enhancing awareness regarding the potential for miscommunication in clinical practice.

## Methods

This cross-sectional study was conducted among dental practitioners with a minimum of 1 year of clinical practice experience in Thailand including Bangkok and other provinces. A total of 78 participants were selected using convenience sampling. All participants provided written consent before the commencement of data collection. The data collection process involved interviews using a questionnaire designed to gather information on 13 selected anatomical terms based on their common usage in dental practice. These terms were determined by a literature review of English-written books, research papers in the field of dentistry, as well as medical dictionaries and dental glossaries. These 13 terms were categorized into two groups: Group 1, comprising 3 terms with clear and unambiguous meanings in scientific literatures, including Mental foramen, Condyle of mandible, and Greater palatine foramen; and Group 2, consisting of 10 terms with ambiguous meanings in scientific literatures, including Mandibular notch, Zygoma, Mylohyoid ridge, Incisive canal, Internal oblique ridge (line), Temporal crest of mandible, Coronoid notch, Sigmoid notch, Mandibular fossa, and External oblique ridge.

**Fig. 1:**
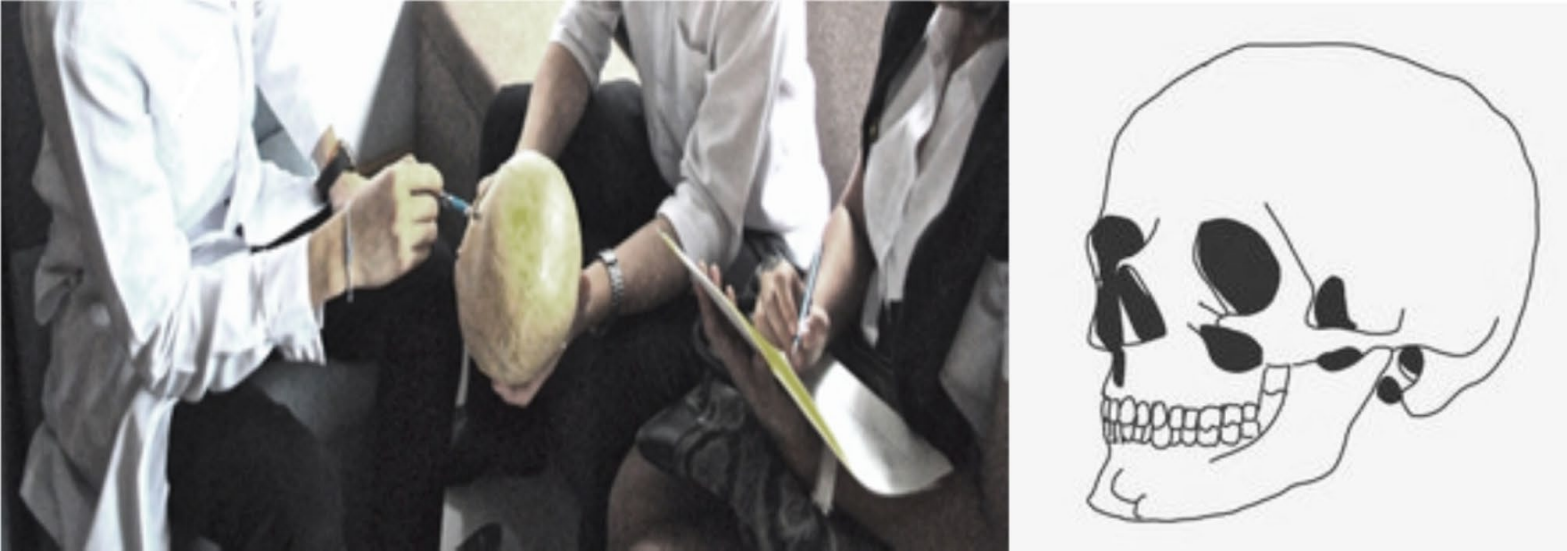
Example of the process (**1**) Dentist located specific term on artificial skull (Left), (**2**) Interviewer recorded on the paper form (Right)

During the interviews, participants were asked to identify the location of each anatomical term based on their understanding and usage on a provided artificial skull by interviewer (PT), and the responses were recorded on the paper containing skull figure (Fig. 1) by data recorder (LM). There was no modification of interviewer’s behaviour and participants’ responses.

In the first part, to ensure the authenticity of responses, the Group 1 terms were taken to verify that participants’ answers were not influenced by ignorance or arbitrary guessing. Eligible participants who mislabelled or incorrectly indicated any of these terms were excluded from the study.

In the second part of the interview, participants were required to identify the Group 2 terms on an artificial skull and provide explanations for each term such as the importance of this location or a landmark for some operations. All data were recorded in the record form (Supplementary 1), and the findings were reported as descriptive statistics by researchers (KC, TA, PG).

This study received approval from the Mahidol Ethical Approval Committee (MU-DT/PY-IRB 2011/059.1209).

## Results

Among the 78 participants, all participating dentists were eligible for the study. The average ages were 43.8 years old (ranging from 28 to 60 years old) with working experiences in dentistry ranging from 5 to 35 years. The majority specialized in prosthetic dentistry (*N* = 28), followed by advanced general dentistry (*N* = 15), oral and maxillofacial surgery (*N* = 10), operative dentistry and endodontics (*N* = 10), oral medicine (*N* = 5), oral and maxillofacial radiology (*N* = 5), and paediatric dentistry (*N* = 5). All participants have already completed their postgraduate studies. While they all graduated with a bachelor’s degree in Dentistry in Thailand, their postgraduate training varied, taking place both in Thailand and internationally in countries such as the United States, the United Kingdom, Germany and Japan.

All participants correctly identified and labelled three anatomical terms with clear clinical significance: condyle of the mandible, mental foramen, and greater palatine foramen. Therefore, no participants were excluded from the study.

Table 1 shows the results for the 10 anatomical terms that have ambiguous meaning in clinical use. Mandibular notch was in two different areas on the skulls: the concave region of the superior border of the ramus (25%) and the concave region of the anterior margin of the ramus (25%), while 33% of participants were unable to define this term. Coronoid notch was frequently confused with mandibular notch and was located at the concave region of the superior border of the ramus (51.32%) and the concave region of the anterior border of the ramus (18.42%), while 13.16% of participants reported not knowing or being unable to define this term. More than three-quarters of participants (77.62%) reported being unable to define the term sigmoid notch, but 17.11% of dental surgeon experts indicated it as the concave region of the superior border of the ramus.

**Table 1:** The response from participants for each indicated location (*N* = 76)

	Indicated location	Number	(%)	
**Mandibular notch**				
	1.Concave region at upper border of ramus	19	25	
	2. Concave region at anterior border of ramus	19	25	
	3. Concave region at lower border of the end of mandible	5	7	
	4. Concave region at inner mandible; above mental foramen	5	7	
	5. Other areas	3	33	
	Not label/Not known this term	25	33	
**Coronoid notch**				
	1.Concave at upper border of ramus	39	51.32	
	2. Concave at anterior border of ramus	14	18.42	
	3.Coronoid process of mandible	12	15.78	
	4.Locate at 1 and 2.	1	1.32	
	Not label/Not known this term	10	13.16	
**Sigmoid notch**				
	1. Concave region at upper border of ramus	13	17.11	
	2. Concave region at lower border at the end of mandible	1	1.32	
	3. Other areas	3	3.95	
	Not label/Not known this term	59	77.62	
**Zygoma**				
	1. Zygomatic bone	46	50.5	
	2. Zygomatic arch	4	5.3	
	3. Zygomatic bone and zygomatic arch	24	31.7	
	4. Locate 1, 2, or 3	1	1.5	
	Not label/Not known this term	1	1.5	
**Mylohyoid ridge**				
	1. Bone crest at mesial of body of mandible and insertion area of mylohyoid ridge	64	84.5	
	2. Other areas	5	6	
	Not label/Not known this term	7	9.5	
**Incisive canal**				
	1. Sphenopalatine nerve pass through canal; opening at incisive foramen	69	90.8	
	2. Canal which inferior alveolar nerve lies	2	2.6	
	3. Locate at 1 and 2	2	2.6	
	4. Other areas	1	1.4	
	Not label/Not known this term	2	2.6	
**Internal oblique ridge (line)**				
	1. Bone crest at inner of coronoid process which pass on medial surface of ramus; insertion of deep tendon of temporal muscle	65	85.5	
	2. Bone crest at medial surface of ramus (Distal of lower third molar)	5	6.5	
	3. Mylohyoid ridge	3	4	
	Not label/Not known this term	3	4	
**Temporal crest of mandible**				
	1. Bone crest at inner of coronoid process which pass on medial surface of ramus; insertion of deep tendon of temporal muscle	2	2.65	
	2. Other areas	9	11.82	
	Not label/Not known this term	65	85.53	
**Mandibular fossa**				
	1. Area at base of skull; part of TMJ	2	2.63	
	2. Deepest concave region of anterior border of ramus	2	2.63	
	**3. Other areas**	26	34.21	
	Not label/Not known this term	46	60.53	
**External oblique ridge**				
	1. Anterior aspect of ridge which ended at the end of coronoid process	38	50	
	2. Bone crest at the end of body of mandible to the middle of ramus	36	47.36	
	3. Bone crest at inner of coronoid process which lies on medial surface of ramus	1	1.32	
	Not label/Not known this term	1	1.32	

Zygoma was located in two different areas, namely the zygomatic bone (50.5%) and the zygomatic bone with zygomatic arch (31.7%). Mylohyoid ridge was indicated at the bone crest, which lies on the medial aspect of the body of the mandible acting as the attachment of the mylohyoid muscle (84.5%), and in other areas or not known (6% and 9.5%, respectively). Incisive canal was labelled on the maxilla area, where the nasopalatine nerve passes through the canal and opens into the incisive foramen by 90.8% of participants. However, a few dentists located this canal in a different area, namely the ramus of the mandible, where the canal was the passing way for the inferior alveolar nerve. Internal oblique ridge (line) was located at the bone crest as the inner side of the coronoid process, which lies on the medial surface of the ramus to the area nearby the distal end of the mandibular third molar by 85.5% of participants. However, 6.5% of participants indicated this term at the medial surface of the ramus, which is the distal end of the mandibular third molar only. In addition, most participants (85.5%) were unable to define the term temporal crest of mandible. Sixty percent of participants were unable to locate the mandibular fossa, and this term was in different areas by about 34% of participants. Moreover, the mandibular fossa was replaced by other terms such as “temporomandibular fossa,” “articular fossa,” “glenoid fossa,” and “condylar fossa” in the interview.

Finally, the external oblique ridge was located at the anterior aspect of the ramus until the end of the coronoid process (50%) or at the bone crest of the distal body of the mandible (47.4%).

## Discussion

The usage of anatomical terms is essential for dental professionals to communicate with each other and to understand the location of various parts of the head and skull. The present study found that some anatomical terms used in the dental clinical setting have different meanings from their definition in the anatomical terminology textbooks. This discrepancy can lead to confusion and miscommunication among dental professionals, especially for those who are just starting their education.

In this study, the terms “Mandibular fossa” or “fossa mandibularis” was defined in the TA as “A prominent depression in the inferior surface of the squamous part of the temporal bone at the base of the zygomatic process in which the condyloid process of the mandible rests” [6]. Naming a fossa or depression based on its articulating structure is a common practice in anatomical terminology. Similar examples can be found in general anatomy, such as the olecranon fossa of the humerus, which accommodates the olecranon of the ulna, the vermian fossa of the occipital bone, which houses part of the inferior cerebellar vermis, and the digastric fossa of the mandible, which serves as the attachment site for the anterior belly of the digastric muscle. Moreover, in a local anaesthesia textbook for dentists [14], another meaning of the term “mandibular fossa” is employed to denote the foramen located on the medial surface of the mandibular ramus, a structure more commonly recognized as the “mandibular foramen”. Surprisingly, the result of this study indicated that very few dentists used “mandibular fossa” in either meaning according to the TA term (2.6%) or in the context of “mandibular foramen” (2.6%). The majority of dentists are unfamiliar with the term (60.5%). This lack of familiarity might be due to the occurrence of alternative terms in dentistry. For the first meaning, other terms like “articular fossa” or “glenoid fossa of the temporal bone” are more commonly used. Additionally, for the second meaning, dentists frequently use the term “mandibular foramen”, rendering the term “mandibular fossa” obsolete. Consequently, “mandibular fossa” is a term that most dentists are unfamiliar with, despite its fundamental relevance to the direct practice of dentistry.

The terms “Mandibular notch,” “Coronoid notch,” and “Sigmoid notch” illustrated the complexity arising from homonyms and synonyms in dental terminology. According to the TA term, “Mandibular notch” is considered synonymous with “Sigmoid notch,” and “Mandibular incisure” or “Incisura Mandibulae” referring to “the concave area at the superior border of the mandibular ramus” [6]. However, in anatomical textbooks designed for dental students, “Mandibular notch” was not only presented in the context of the TA term but also described as a synonym for “Coronoid notch” [15], a term that was not included in the TA. Furthermore, in various dental textbooks, “Mandibular notch” was homonymously defined as “the concave area at the anterior border of the mandibular ramus“ [16] and was synonymous with “coronoid notch” in this context [14, 17]. This confusion between “Mandibular notch” and “Coronoid notch” held considerable significance within dentistry, emphasizing the variability in the use and interpretation of these terms within the same professional community. The present study revealed the considerable variability in the usage and understanding of the terms “Mandibular notch,” “Coronoid notch,” and “Sigmoid notch” among dentists, especially within specific dental subspecialties like oral and maxillofacial surgery. The research showed that even among dental professionals, there were discrepancies in the interpretation of these terms. Notably, the term “Sigmoid notch,” as per the TA term, was rarely used in dental literature and was unfamiliar to most dentists. Instead, dentists tended to use alternative terms such as “Coronoid notch” or “Mandibular notch,” leading to potential misunderstandings, especially in interdisciplinary communication within dentistry. This aligned with the present findings, which revealed that dentists described both terms in similar contexts. However, the term “Sigmoid notch,” which was an anatomical term, was rarely encountered in dental literature, making most dentists unfamiliar with its meaning, despite its relevance to dentistry-related contexts. Moreover, among the dentists who did specify the meaning of “Sigmoid notch” according to the TA term (17.1%), nearly all reported by participants who specialized in oral and maxillofacial surgery, reflecting that even within the dental profession, there were variations in the use of terminology among different subspecialties.

The present study further demonstrated that the terms “Mandibular notch,” “Coronoid notch,” and “Sigmoid notch” were used interchangeably, not only in describing the “anterior” or “superior” border of the mandibular ramus but also in other meanings that were not consistently documented in written literatures. This inconsistency and interchangeability in the usage of these terms emphasized the need for standardized and precise anatomical terminology, especially in the field of dentistry, to ensure clear communication and understanding among professionals, regardless of their subspecialties.

The emergence of broader meanings for specific terms over time is a common phenomenon in language evolution. This evolution is often driven by the necessity to describe specific anatomical regions more precisely due to the development of specialized knowledge within various fields. Consequently, new anatomical terms may be coincided to meet the demands of evolving academic disciplines. This process can lead to the creation of new meanings for existing terms, especially in response to the changing requirements of specific fields. Over time, as these new meanings become widely accepted within particular disciplines, they may not pose issues [11, 18]. For instance, the use of the term “sigmoid notch” in oral and maxillofacial surgery might not cause confusion within that specific subspecialty. However, challenges arise when interdisciplinary communication occurs, even among different subspecialties within dentistry. In these situations, professionals might use different terms, such as “coronoid notch” or “mandibular notch,” leading to misunderstandings and miscommunications. Therefore, it remains essential to establish precise and standardized anatomical terminology to facilitate effective communication and understanding across various dental subspecialties and, more broadly, within the field of healthcare.

“The concave area at the anterior border of the mandibular ramus” was not only referred to as the Mandibular notch or Coronoid notch but the present study demonstrated that most dentists were also familiar with another term, the External oblique ridge. Interestingly, even though almost all dentists described this term in a similar location, they defined its boundaries differently. Half of them specified the inferior end of the external oblique ridge in the area distal to the last molar along the anterior border of the ramus. However, the other dentists (47.4%) extended the definition of the inferior end of the external oblique ridge to the area encompassing the second premolar. The latter group primarily consisted of oral and maxillofacial radiologists. Based on their experience with radiographic images, they observed the radiopaque nature of the external oblique ridge extending to the second premolar. This variation in descriptions supported the differences in interpretations of the term, which depend on the expertise and specialized focus of each dental subspecialty.

The terms “Internal oblique ridge” or “internal oblique line” are another example of the creation of new terms to provide to the specific needs of dental contexts. These terms refer to the bony ridge on the medial surface of the mandibular ramus, extending from the coronoid process to the posterior area of the last molar tooth [5, 10,19, 20]. This ridge serves as the distal attachment of the deep tendon of the temporalis muscle, which holds clinical significance in dentistry. Surprisingly, this specific region is not mentioned in the TA or general anatomical literature. According to Cunningham’s anatomy textbook [21], it is briefly described as a “blunt ridge”. Therefore, the term “Internal oblique ridge” was coincided to facilitate communication within the dental field and could only be found in the dental textbooks such as Jorgensen and Hayden’s textbook [22]. This illustrated the necessity of creating specialized terms to address specific anatomical features significant in dental practice, even if they were not extensively covered in general anatomical literature. Additionally, in dental textbooks, the term “Internal oblique ridge” could also be found under such different names as the “temporal crest of the mandible” [7] and “mandibular temporal crest” [8]. However, the study revealed that the majority of dentists were more familiar with the term “Internal oblique ridge”, as opposed to terms like “temporal crest” when referring to the bony ridge serving as the distal attachment of the deep tendon of the temporalis muscle. This indicated a clearer preference for the term “Internal oblique ridge” among dentists, showcasing the importance of standardized terminology for effective communication within the dental field. The study demonstrated that even though specialized terms were coincided within specific fields and documented in written literatures, they might not gain popularity in clinical usage and could fade away over time. When these terms were introduced, they could lead to confusion in meaning, as evidenced by the study results. This underscored the importance of caution and precision in using terminologies, both in educational contexts and clinical communication, especially in interdisciplinary communication within specialized fields.

The results were in line with the previous studies which terminologies need to be expanded to the terms that used by clinicians and should accommodate with both clinical and anatomical works [23]. This highlights the importance of clear and consistent communication within and across medical community [9, 24] included dental specialties, as well as the need for standardized terminology in the field. However, there were limitations in this study to be further improved. First, the study was conducted with a small sample size and a specific sampling method. Although the interviewees had experience in training both in Thailand and internationally, the limited sample size raises concerns about the generalizability of the data. A multi-centre study should be warranted in the conclusion. However, the results of this study could help dental educators become aware of this issue. Second, the selection of anatomical landmarks was limited to those in previous reports; expanding the terminology specific to the dental field might improve the study by focusing on dental communication. Third, the study design limited opportunities for data analysis. Further studies should improve data collection and provide more thorough analysis. However, this field in dentistry lacks evidence, so this study highlights the issue of confusion among academics that needs to be addressed.

Furthermore, the present study highlighted the emergence of new and broader meanings for existing anatomical terms over time. This phenomenon was common in language evolution, where terms expanded in meaning based on the specific needs and developments within different scientific disciplines. The new terminology has not been defined or approved by international committee or clarified the detail of terminology, while commonly using in dental field only. To our concern, the synonym and homonyms could make students confused due to the various terms. Moreover, dental textbooks had different terms to indicate various anatomical areas. This problem could affect students who just start in dental study would be confused in learning, practicing, or using those term without awareness among students and professors or specialists [11]. Overall, the findings of this study suggest that there is a need to systematically revise the usage of anatomical terms between anatomists and clinicians to reduce ambiguity and miscommunication in the dental clinical setting [25]. Standardizing the terminology used in dental education and clinical practice would also benefit dental professionals and students by providing clear and consistent communication in the field [5, 10, 26]. In conclusion, the study emphasizes the importance of clear and consistent terminology in the field of dentistry and the need for effective communication, especially when dealing with interdisciplinary or subspecialty interactions.

This issue could lead to confusion and miscommunication among dental professionals and students, especially those who are just starting their dental education [27]. We recommend that this problem be addressed by increasing awareness among dental professionals and students and by revising the usage of anatomical terms in a more systematic and standardized manner [28, 29]. While several terms were compiled in TA, they were still insufficient to keep pace with the rapid academic advancements occurring in various fields today. This would lead to better communication and collaboration among dental professionals and between dental and medical professionals, improving patient care and outcomes.

## Conclusion

In conclusion, our study highlights the philological ambiguity of anatomical terms among different specialties of dentistry. The usage of anatomical terms varies among specialties, with some terms having different meanings or being used to describe different locations than their original definitions.

### Electronic supplementary material

Below is the link to the electronic supplementary material.

Supplementary Material 1

## Data Availability

The datasets generated and analysed during the current study are not publicly available due to paper forms which archived in the collection but are available from the corresponding author on reasonable request.

## Abbreviations

TA: Terminologia anatomica
