# Supplementary material for: Exploring the ambiguity in the anatomical terminology among Dental professionals
Source: BMC Med Educ. 2024 Aug 22;24:904. doi: 10.1186/s12909-024-05878-1 (PMC11342683; doi:10.1186/s12909-024-05878-1)

**แบบบันทึกผลการสัมภาษณ์**

**โครงการวิจัย ความไม่ลงรอยเชิงนิรุกติศาสตร์ของศัพท์กายวิภาคศาสตร์ในการใช้ทางคลินิก**

**Philological ambiguity : Anatomical – clinical discrepancy**

❒ ไม่ทราบความหมายของคำศัพท์………………………………………………………………………………………………………


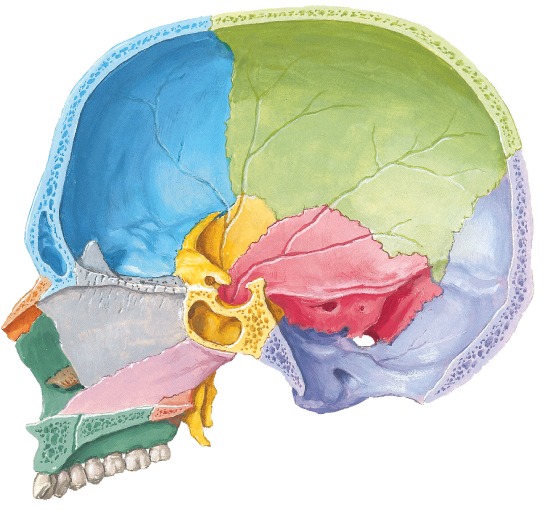
อื่นๆ ................................................................. ............................................................ ............................................................ ................


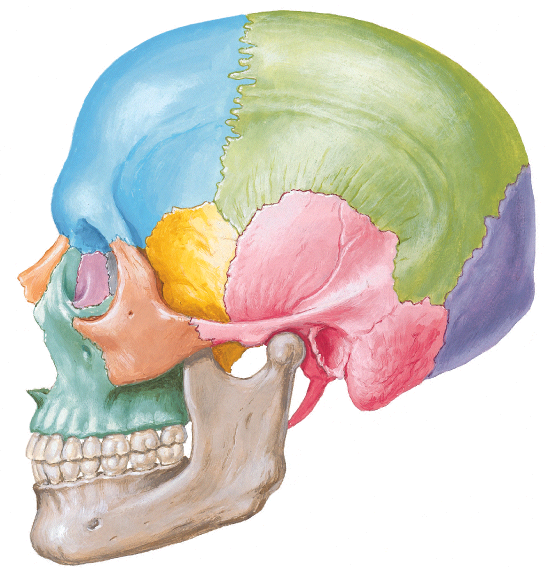


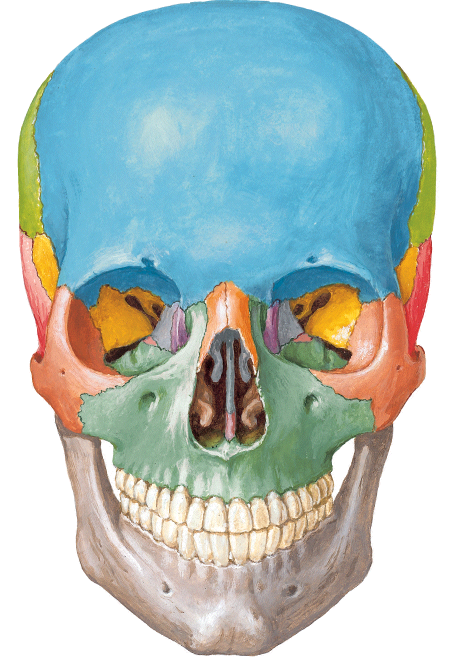


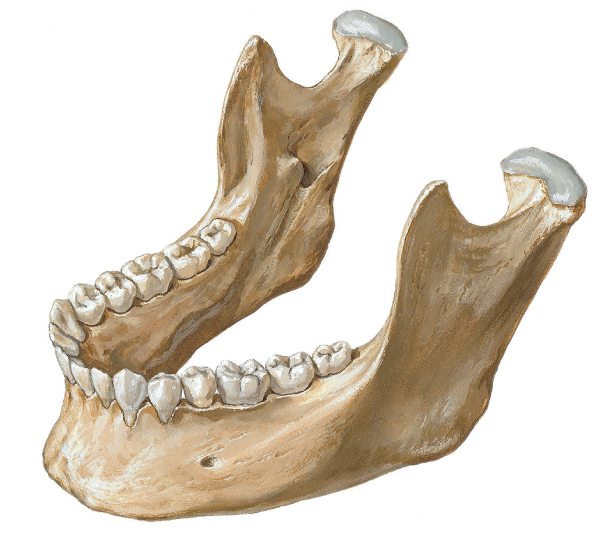


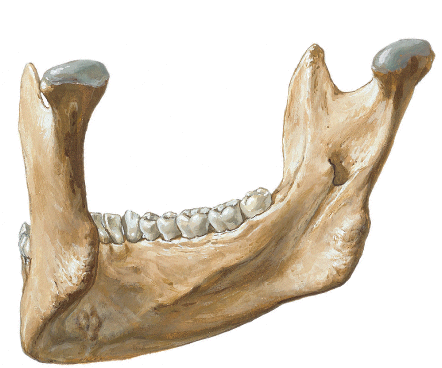


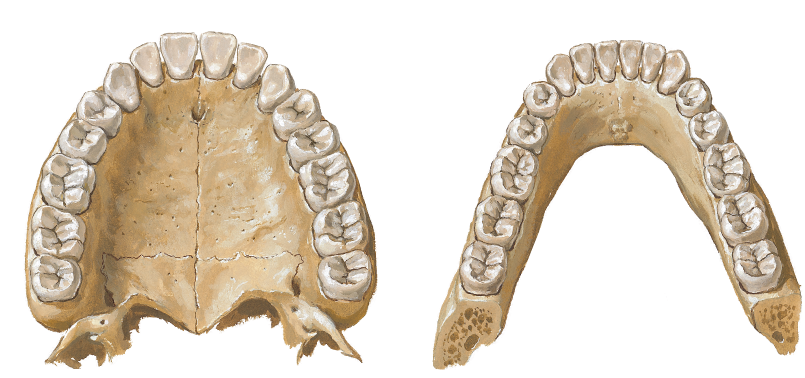

Supplement: Supplementary file 1 — Supplementary Material 1 [file 12909_2024_5878_MOESM1_ESM.doc]
